# Central nervous system-derived extracellular vesicles: the next generation of neural circulating biomarkers?

**DOI:** 10.1186/s40035-024-00418-9

**Published:** 2024-06-19

**Authors:** Rocío del Carmen Bravo-Miana, Jone Karmele Arizaga-Echebarria, David Otaegui

**Affiliations:** 1 Multiple Sclerosis Group, Neuroscience Area, Biodonostia Health Research Institute, San Sebastián, 20014 Spain; 2 Centro de Investigación Biomédica en Red de Enfermedades Neurodegenerativas, Instituto de Salud Carlos III, Madrid, 28029 Spain

**Keywords:** Extracellular vesicles, Circulating biomarkers, Central nervous system-derived EVs, Neural-derived EVs, Brain-derived EVs, Cerebrospinal fluid EVs, Plasma EVs, Neurodegenerative diseases

## Abstract

The central nervous system (CNS) is integrated by glial and neuronal cells, and both release extracellular vesicles (EVs) that participate in CNS homeostasis. EVs could be one of the best candidates to operate as nanosized biological platforms for analysing multidimensional bioactive cargos, which are protected during systemic circulation of EVs. Having a window into the molecular level processes that are happening in the CNS could open a new avenue in CNS research. This raises a particular point of interest: can CNS-derived EVs in blood serve as circulating biomarkers that reflect the pathological status of neurological diseases? L1 cell adhesion molecule (L1CAM) is a widely reported biomarker to identify CNS-derived EVs in peripheral blood. However, it has been demonstrated that L1CAM is also expressed outside the CNS. Given that principal data related to neurodegenerative diseases, such as multiple sclerosis, amyotrophic lateral sclerosis, Parkinson’s disease and Alzheimer’s disease were obtained using L1CAM-positive EVs, efforts to overcome present challenges related to its specificity are required. In this sense, other surface biomarkers for CNS-derived EVs, such as glutamate aspartate transporter (GLAST) and myelin oligodendrocyte glycoprotein (MOG), among others, have started to be used. Establishing a panel of EV biomarkers to analyse CNS-derived EVs in blood could increase the specificity and sensitivity necessary for these types of studies. This review covers the main evidence related to CNS-derived EVs in cerebrospinal fluid and blood samples of patients with neurological diseases, focusing on the reported biomarkers and the technical possibilities for their isolation. EVs are emerging as a mirror of brain physiopathology, reflecting both localized and systemic changes. Therefore, when the technical hindrances for EV research and clinical applications are overcome, novel disease-specific panels of EV biomarkers would be discovered to facilitate transformation from traditional medicine to personalized medicine.

## Introduction

The high social and economic impacts of pathologies associated with the central nervous system (CNS) are one of the major problems in developed countries, associated with disabilities and high prevalence in the elderly population [1]. There is an urgent need for non-invasive and easily detectable biomarkers that could identify the disease at early onset (asymptomatic individuals), detect or confirm the presence of a disease and its specific subtype (diagnostic), identify the low/high-risk course of the disease or disease recurrence (disease activity), monitor the response to disease-modifying therapies (treatment response), and track the disease progression (follow-up), helping the field to move towards personalized medicine.

The CNS is integrated by glial (astrocytes, oligodendrocytes and microglia) and neuronal cells, which act in concert to maintain its homeostasis. The intercellular communications among these cells are produced through classical cell-to-cell interactions and via several molecules released in a soluble form or packaged into membrane-enclosed vesicles called extracellular vesicles (EVs) that are released to the extracellular milieu and identified in biological fluids. The cargos inside EVs include RNAs, DNAs, metabolites, lipids, and proteins [2]. These EVs have the ability to concentrate their cell-specific cargos, allowing the identification of less abundant molecules when compared to the total biofluid, and protecting them from proteasas, nucleasas, and other systemic enzymes during their systemic circulation [3]. However, a dilemma in studies of circulating EVs as biomarkers is to identify the putative origin of the EVs. In this sense, EV membrane proteins can function as a biomarker, but also as a potential “hook” to trace and enrich certain types of EVs from the rest.

This scenario leads us to a particular point of interest: would CNS-derived EVs in blood serve as circulating biomarkers that reflect the pathological status of neurological diseases? In order to answer this question, the aim of this review is to highlight the presence and relevance of CNS-derived EVs in cerebrospinal fluid (CSF) and blood samples of patients with neurological diseases, focusing on the reported current biomarkers and the technical possibilities for their isolation.

The most used biomarker for neuron-derived EVs is L1 cell adhesion molecule (L1CAM) that was proposed by Shi et al., 2014 and then adopted by many other researchers [4, 5]. However, it was demonstrated later that L1CAM is also expressed in other CNS-cells (such as oligodendrocytes) and peripheral nervous system cells (such as Schwann cells). At the same time, it was evidenced outside the nervous system in immune cells (monocytes, T and B cells), melanocytes, kidney tubule epithelial, intestinal crypt cells, and certain types of endothelial and cancer cells [6]. At this point, it is important to differentiate between the expression of a protein in a tissue and its consequent presence in EVs. In that sense, there is no currently evidence to accurately discern whether the EVs carrying the studied biomarkers come only from the CNS or from other tissues in which they are also expressed. On the other hand, Norman and coworkers [7] demonstrated that in human plasma or CSF, L1CAM could be present in its cleaved form, alternatively spliced form or both soluble forms. Besides, they reported cross-reactivity of at least one L1CAM antibody (UJ127 clone) with α-synuclein, a protein usually tested as an EV biomarker of Parkinson’s disease (PD). Then, given these controversies, Gomes and Witwer [6] published a systematic review emphasizing the necessity to consider combinations of different isolation techniques, several controls, and the use of antibodies against extracellular and internal epitopes to exclude artefacts of binding or aggregation in the different EV experiments.

### Overview of EVs

EVs are a heterogeneous population of lipid bilayer-delimited nanoparticles that are released by cells into the extracellular space [8]. The first observation about the presence of vesicles in biological fluids was made in 1946, when Chargaff and West studied the anticoagulant properties of platelet-derived particles in plasma [9]. However, until 1987, EVs were still considered a waste disposal system of unnecessary transmembrane proteins [10]. In 1998, due to the ability of EVs to prompt signaling pathways in recipient cells, transporting the functional major histocompatibility complex and T-cell co-stimulatory molecules, the interest of scientists in this field was renewed [11]. EVs can be classified into three different types according to their biogenesis pathway: exosomes, microvesicles, and apoptotic vesicles. Exosomes are released after the fusion of a late endosome or multivesicular body with the plasma membrane (PM), while microvesicles evaginate directly from specific microdomains of the PM. Cells undergoing apoptosis form apoptotic bodies or vesicles by PM blebbing or protrusion formation. The size ranges of exosomes (30–150 nm), microvesicles (50–1000 nm), and apoptotic vesicles (100–5000 nm) overlap [12]. Nevertheless, the different biogenesis pathways of EV subtypes cannot be fully separated according to biophysical characteristics (size and density) or structural and molecular components [13]. In this context, the International Society of Extracellular Vesicles (ISEV) founded in 2011 has reached a consensus on the use of EVs as a generic term for all vesicles produced by cells [14]. Moreover, the co-isolation of different subsets of non-EV particles (lipoproteins, enveloped viruses, exomeres) is important to consider [15], especially when they exert different pathophysiological roles in recipient cells or are studied with translational purposes. Cocozza et al. (2019) [16] have summarized the most commonly used isolation techniques together with their recovery versus specificity performance. Precipitation and filter concentration are considered to provide high recovery of EVs but with low specificity, since they enrich EVs along with other particles and many other secreted molecules. Techniques with medium recovery and specificity, such as differential ultracentrifugation and size exclusion chromatography (SEC), enable the enrichment of EVs based on physical characteristics such as size and/or weight. Particularly, EV enrichment by SEC has gained a lot of visibility in recent years considering that soluble components and smaller lipoproteins remain longer in the column, allowing EV separation from them. Finally, density gradients, asymmetric flow field-flow fractionation and immunoprecipitation are considered to provide low recovery of EVs but with high specificity. In this regard, non-lipidic structures could be easily separated from EVs by density gradients/cushions. On the other hand, the use of specific antibodies that bind a given surface protein of EVs achieves the most specific separation. To note, combinations of them improve specificity but limit the yield of preparations. Given that EVs are regarded as one of the most ubiquitous particles, their roles in several mechanisms across a wide spectrum of pathologies have been studied to date.

### Roles of EVs in the CNS

**Fig. 1:**
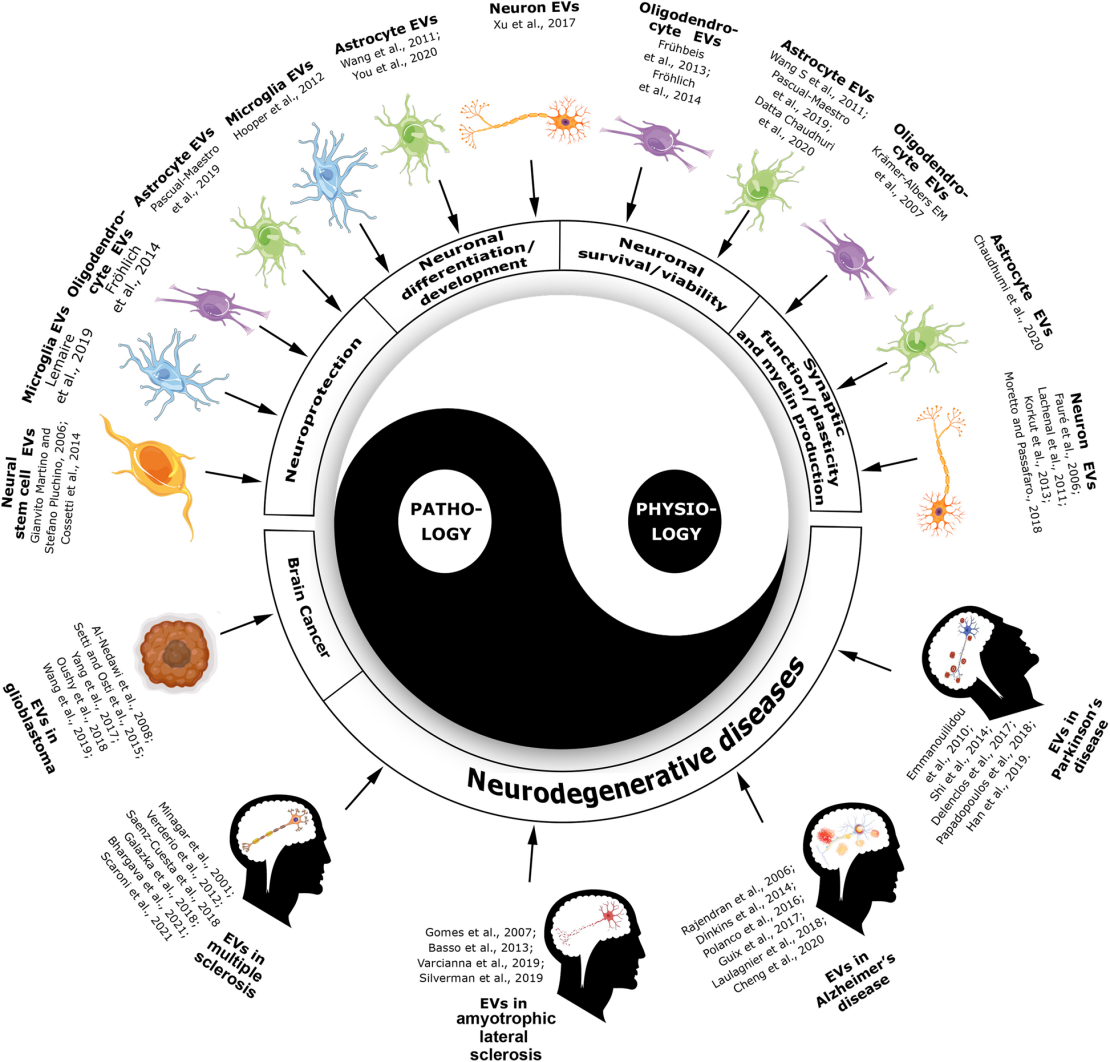
The “yin-yang effect” of EVs in the CNS. EVs from neurons, neural stem cells and glial cells (astrocytes, oligodendrocytes and microglia) contribute to physiological processes as neuroprotection; neuronal differentiation and development; neuronal survival and viability; and synaptic function, synaptic plasticity and myelin production. On the other hand, EVs participate in pathological mechanisms such as brain cancer and neurodegenerative diseases (multiple sclerosis, amyotrophic lateral sclerosis, Alzheimer and Parkinson diseases). Other contributions of EVs in pathological conditions such as stroke, spinal cord injury, traumatic brain injury, and Huntington’s disease, have been less reported. To be noted, the experiments to prove the physiological activities of EVs were carried out using in vitro systems and some conclusions were suggested by the authors rather than experimentally proven. At the same time, contributions of EVs to CNS diseases were mainly investigated using patient samples

EVs play an important role in intercellular communication in the CNS [17–19]. There has been evidence for different roles of EVs in synaptic and neuron-glia communication as well as in communications among different glial cells. CNS-derived EVs have been implicated in synaptic function, synaptic plasticity and myelin production; neuronal survival and viability; neuronal differentiation, maturation and development; as well as neuroprotection, among their physiological roles in the CNS (Fig. 1). EVs also participate in the bidirectional crosstalk among tumor and non-neoplastic cells in the brain, contributing to tumor development [20]. Moreover, EVs have been found to play a pathological role in modulating neuroinflammatory and neurodegenerative processes in patients with multiple sclerosis (MS) [20, 21], amyotrophic lateral sclerosis (ALS), PD, and Alzheimer’s disease (AD), among others [22]. A systematic review by Long and colleagues [23] suggests that the principal contributions of EV studies in the field of neuroscience were made to AD research. Then, EV studies in PD and MS are promising and progressing areas. Conversely, other EV-related diseases with less investigation such as stroke, spinal cord injury and traumatic brain injury are emerging areas. Finally, it is essential to consider that many experiments were performed in vitro, providing a limited understanding of what occurs in living animals or humans, as in the case of EV uptake experiments [19]. Nevertheless, the physio/pathological implications of EVs have opened up avenues for the development of neuroprotective and neuroreparative therapeutic approaches and the discovery of novel and specific biomarkers to achieve personalized medicine.

### CNS-derived EVs as circulating biomarkers in CSF and blood

CSF typically consists of water, electrolytes, metabolites, neurotransmitters, hormones, proteins, and a low number of cells. The CSF performs vital functions such as nourishment, waste removal, and protection. The brain is specifically sealed off from peripheral fluid exchange by tightly packed endothelial and epithelial juctions that generate barriers sealing the CSF and brain or spinal cord microenvironment from bloodstream and peripheral extracellular fluid [24]. The blood-brain barrier and the blood-CSF barrier, commonly identified as the blood-CNS barrier (BCNSB), regulate the trafficking of solutes between the blood and the CNS. The CSF contains roughly 5 cells per microliter and has 200 times less protein than the plasma. CSF is considered the most promising body fluid for the discovery of neural protein biomarkers [25, 26]. However, in individuals with BCNSB dysfunction, about 80% of the overall protein content in the CSF originates from the plasma through filtration across the cell barrier, with albumin as the main contributor [27]. In addition, a critical pre-analytical factor to consider is the potential blood contamination of CSF samples during lumbar puncture. Since blood contains high protein levels, the presence of only a small amount of blood in the CSF can significantly affect the CSF concentrations of proteins, especially blood-derived proteins such as hemoglobin, catalase, peroxiredoxin and carbonic anhydrase I [25, 28]. A consensus protocol has established that CSF samples with blood contamination of more than 500 erythrocytes/µl should not be used for biomarker studies [29].

One of the first reports of EVs from CSF (CSF-EVs) was published by Street et al. in 2012 [30]. The authors highlighted the need for a refinement of isolation techniques to obtain EVs from this biofluid to reduce sample-to-sample variability. Another challenge is the required pooling of samples to obtain sufficient starting material, which results in the loss of individual variations among patients, making it difficult to identify specific differences and correlations among biomarker levels [31]. Finally, the main obstacle is the invasive procedure of CSF collection, making it difficult to perform repeated sampling over time.

On the other hand, one of the most studied sources of EVs is blood, as it is easily accessible with a minimally invasive procedure [32]. However, it is challenging to find specific CNS-derived EVs that are present in lower concentrations in blood by crossing the blood-CSF barrier to the systemic circulation [26]. The research on CNS-derived EVs as blood-circulating biomarkers is still in development, and more investigations are required to enhance the sensitivity and specificity of EV isolation techniques to enrich and trace this particular EV subpopulation in blood. In the following sections, biomarkers for the isolation of CNS-derived EVs from CSF and blood samples of patients with MS, ALS, PD, and AD will be recapitulated.

### MS

MS is a chronic inflammatory demyelinating disease of the CNS that affects 2–3 million people worldwide. The highest prevalence of MS occurs between the ages of 20 and 40, making MS the most common cause of disability in early adult life, with a huge economic and life quality impact [33, 34]. The progression of disability in MS could be illustrated in four stages for most of the patients (about 85%): the presymptomatic phase before the appearance of any clinical symptom, the clinically isolated syndrome (CIS), the relapsing-remitting (RRMS), and the secondary progressive phase of MS [35, 36]. For the remaining 10%–15% of patients, the disease is associated with worsening of symptoms from the onset, known as primary progressive MS (PPMS) [37]. Currently, diagnosis of the disease is made according to the McDonald Criteria [38] based on clinical examination and biomarkers, such as magnetic resonance imaging (MRI) scans and oligoclonal bands in the CSF.

#### CSF-EVs

Lee and colleagues (2016) [39] conducted an analysis of CSF-EVs obtained through differential ultracentrifugation, revealing a specific enrichment of fibronectin in EVs isolated from MS patients. The aggregation of this protein has been identified to play a potential role in the pathogenesis of MS due to its association with impaired remyelination processes. Besides, CSF-EV pools from RRMS patients with respect to idiopathic intracranial hypertension patients showed enrichment of specific proteins such as plasma kallikrein and apolipoprotein E4 in EVs compared to cell-free total CSF. However, kallikrein could not specifically distinguish RRMS patients, so it is uncertain if this enrichment is disease- or CNS-specific. Apolipoprotein E was highlighted since it has been found in EVs in neurological diseases including MS and AD [40]. Geraci et al. (2018) [41] have shown a trend of higher amount of EVs obtained by ultracentrifugation of CSF samples from patients with progressive MS and CIS, compared to other inflammatory and non-inflammatory neurological disorders. However, the CSF-EV concentration did not offer the opportunity to distinguish MS from other neurological diseases. Besides, they focused on the clinico-radiological paradox and highlighted that the importance of some biomarkers is to indicate disease activity even in the absence of visible lesions on MRI scans or clinical symptoms. Then, Gelibter et al. (2021) [42] reported that myeloid vesicles positive for isolectin B4 (IB4, a biomarker that cannot discriminate between microglia and infiltrating macrophages) in fresh CSF, were increased in neuroinflammatory patients compared to the neurodegenerative and control groups. Furthermore, the level of these vesicles was higher in patients with an increased number of enhancing lesions and greater disease activity prior to their diagnosis. Besides, the same group demonstrated that the IB4-myeloid EVs may function as a prognostic risk factor for CIS after a first demyelinating event [43].

#### Blood-EVs

EVs from blood (blood-EVs) are potential biomarkers in MS disease. Published articles related to CNS-derived EVs in the blood are summarized in Table 1.

**Table 1:** CNS-derived EVs in the blood of multiple sclerosis patients

EV-type / Source	PAT / EV-enrichment	EV surface marker	CT	Studied Analytes	Reference
Neuron-derived EVs / Plasma	EDTA Plasma separator tube − 3000 rpm 15 min at RT / 500 µl plasma - Thrombin 30 min at RT − 4000× *g* 20 min at 4 °C / ExoQuick (System Biosciences) followed by 1500× *g* 20 min at 4 °C	L1CAM	NTA	Synaptopodin, Synaptophysin	[44]
Astrocyte-derived EVs / Plasma		GLAST		C1q, C3, C3b/iC3b, C4, C5, C5a, C9, Factor B and Factor H	
Microglia/infiltrating macrophage EVs / Plasma	EDTA Plasma separator tube − 1500× *g* 15 min / 500 µl plasma - Thrombin 5 min − 3000× *g* 5 min at RT / ExoQuick (System Biosciences) followed by 1500× *g* 30 min at 4 °C	IB4 (biotinylated isolectin B4)	TRP WB	let-7b-5p and miR-150-5p	[45]
Oligodendrocyte-derived EVs / Serum	Serum separator tube − 1500× *g* 10 min / 250 µl serum 3000× *g* for 30 min at 4 °C / ExoQuick (System Bioscience) followed by 1500× *g* for 30 min at 4 °C	MOG	NTA TEM WB	MBP	[46]

*PAT *Preanalytical technique, *CT* Characterization technique of EVs, *RT *Room temperature

As mentioned, L1CAM has been used to isolate neuron-derived EVs since 2014, but in the MS field it was not used until 2021, when Pavan Bhargava and co-authors [44] used L1CAM and glutamate aspartate transporter (GLAST) to isolate putative neuron- and astrocyte-derived EVs, respectively, by immunoisolation from the plasma of MS patients. However, they did not find a difference in L1CAM^+^ EV levels between MS patients and controls. Nonetheless, the levels of synaptopodin and synaptophysin were markedly decreased in MS patients (RRMS and progressive MS) vs. controls, without differences between MS subtypes. At the same time, the synaptopodin and synaptophysin levels were inversely correlated with the age of control samples, supporting the relation of this finding with synaptic loss. Regarding GLAST^+^ EVs, a higher concentration has been found in controls vs. MS patients. In addition, components of the early (C1q, C3, C3b/iC3b) and late (C5, C5a) complement cascades and the inhibitor Factor H are increased in GLAST^+^ EVs in plasma from MS patients vs. controls. However, these findings did not align with the results obtained in total EVs or in unprocessed plasma, emphasizing the importance of analysing specific EV subpopulations. Another important finding was the inverse correlation between synaptic proteins in L1CAM^+^ EVs and multiple complement components in GLAST^+^ EVs in MS patients, reflecting a possible pathogenic mechanism. In the study of Scaroni et al. (2022) [45], the IB4^+^ EVs extracted from the total EVs exhibited enrichment of the microglial biomarker TMEM119 than the total EVs, and showed almost no presence of L1CAM, which was present in total EVs. On the other hand, they found elevated levels of miR-150-5p and reduced levels of let-7b-5p in IB4^+^ EVs obtained from cognitively impaired MS patients compared to cognitively preserved MS patients. However, no differences were observed between RRMS and progressive MS patients. To note, the IB4 marker cannot discriminate between microglia and infiltrating macrophages [42]. Another protein that can be employed for EV isolation through immunocapture is myelin oligodendrocyte glycoprotein (MOG), a key component of myelin. Agliardi and coworkers (2023) [46] used MOG to isolate putative oligodendrocyte-derived EVs, and then measured myelin basic protein (MBP), another myelin protein component. They found that MBP was increased in MOG^+^ EVs obtained from serum of MS patients (CIS, RRMS and PPMS subtypes) compared to healthy controls. Additionally, MBP levels were increased in MOG^+^ EVs from PPMS compared to RRMS and CIS. Finally, they observed a positive correlation between the MBP concentration in MOG^+^ EVs and disease severity of MS patients, as measured by the expanded disability status scale and the MS severity score. Given the heterogeneity observed in MS patients, early stratification based on predictions of disease severity will have a huge impact on therapeutic decisions.

### ALS

ALS is a neurodegenerative and devastating proteinopathy, characterized by the loss of cortical, bulbar and spinal motor neurons and a disability of skeletal muscles. This disease presents a limited life expectancy of 2–5 years on average following diagnosis. It is imperative to discover biomarkers for early prediction. EVs are related to ALS and other neurodegenerative disorders by participating in the propagation of misfolded proteins during intercellular crosstalk. Subsequently, these accumulations have the potential to disrupt the regular functioning of neurons, ultimately leading to their demise. While there is extensive support for this hypothesis through in vitro studies, evidence from in vivo studies remains limited [47]. Under pathological conditions, transactive response DNA binding protein (TDP-43), a widely reported protein related to ALS, is translocated from the nucleus to the cytoplasm and then modified through several posttranslational modifications contributing to its misfolding and aggregation.

#### CSF-EVs

Ding and colleagues (2015) reported that full-length TDP-43 (43 kDa) and its C-terminal fragments (35 and 25 kDa) are enriched in the CSF-EVs from ALS patients compared to controls [48]. Previously, another research group indicated limited role of free TDP-43 as a diagnostic tool in ALS patients, as the total TDP-43 in the CSF could predominantly originate from blood. However, TDP-43 measurements might be crucial for tracking the therapeutic effects of disease-modifying therapies focused on this protein [49]. Thompson et al. (2020) [50] demonstrated alterations in the proteomic profile of CSF-EVs from ALS patients, which are associated with protein homeostatic mechanisms that were previously only identified in pathological tissues. The authors also put special emphasis on the technical challenges arising from the low abundance of EVs in CSF, highlighting the significance of CSF starting volume. In a previous study [51] on CSF-EVs from ALS patients, researchers only identified 334 proteins using 3.5 ml of CSF sample, in contrast to the 1020 proteins identified in the reported subsequent study [50] from 7.2 ml sample. The proteins differentially enriched in CSF-EVs of patients with ALS include Bleomycin hydrolase, a protease homologous to the proteasome [50], and the nucleolar complex protein 2 homolog [51], an inhibitor of histone acetyltransferase activity. Interestingly, the latter protein showed decreased expression in the nucleus of motor neurons. Therefore, further research is needed to analyse the potential relationship among its decreased expression, translocation to the cytoplasm and release into EVs to the extracellular environment. Finally, Sjoqvist and Otake (2022) employed a proximity extension assay of the cardiovascular III-panel to examine potential biomarkers in the CSF and the CSF-EVs in ALS patients. The results showed no significant differences in the levels of proteins in CSF-EVs between patients and controls, despite a slight tendency towards downregulation of Perlecan, a protein associated with the formation of synaptic structures and the clearing of misfolded proteins [52].

#### Blood-EVs

Up to date, studies focused on biomarkers for the diagnosis of ALS have included spinal cord markers, neurofilament subunits, electrical impedance myography, oxidative stress markers, neurotrophin receptors, and inflammation markers, among others [53]. Although it has been established that L1CAM is not a specific marker of CNS-derived EVs, it has been employed as a biomarker in ALS (Table 2).

**Table 2:** CNS-derived EVs in the blood of amyotrophic lateral sclerosis patients

EV-type / Source	PAT / EV-enrichment	EV surface marker	CT	Studied Analytes	Reference
Neuron-derived EVs / Plasma	EDTA Plasma separator tube / 2000 µl plasma - PEG-based precipitation − 1500× *g* 30 min at 4 °C	L1CAM	Flow cytometry DLS	microRNAs	[54]
Neuron-derived EVs / Plasma	EDTA Plasma separator tube − 2000× *g* 15 min 4 °C / 500 µl plasma - Thrombin 30 min at RT − 4500× *g* 20 min at 4 °C / ExoQuick (System Bioscience) followed by 1500× *g* for 20 min at 4 °C	L1CAM	NTA ELISA Array	microRNAs	[55]
Neuron-derived EVs / Plasma	EDTA Plasma separator tube − 2000× *g* 15 min at 4 °C / 500 µl plasma - Thrombin 30 min at RT − 4000× *g* 20 min at 4 °C / PEG-based precipitation	L1CAM	-	microRNAs	[56]

*PAT *Preanalytical technique, *CT* Characterization technique of EVs, *RT* Room temperature, *PEG* Polyethylene glycol

As was revised and highlighted by Barbo and Ravnik-Glavač (2023) [53], CNS-derived EVs in blood could be the best approach to identify robust diagnostic and prognostic EV biomarkers in ALS. Studies have identified deregulated miRNAs in L1CAM^+^ EVs, suggesting their potential as biomarkers for ALS [54–56]. In the clinic, it would be ideal to correlate symptom onset, symptom duration, genetics, disease progression, and the prognostic and diagnostic potential of EV-miRNAs differentially identified [56]. Banack et al. (2020) [55] reported differentiation of ALS from ALS-mimic diseases, such as motor neuron disease, using a panel of 8 miRNAs in EVs as biomarkers. They further found in a larger cohort that 5 of these 8 miRNAs (hsa-miR-4454, hsa-miR-151a-5p, hsa-miR-146a-5p, hsa-miR-10b-5p, and hsa-miR-29b-3p) could be used as biomarkers for ALS [56]. These studies showed that there is a fingerprint of EV-miRNAs that could be specifically associated with ALS.

### PD

PD is the second most common neurodegenerative disorder worldwide. This disease presents an irreversible loss of dopaminergic motor neurons in the substantia nigra and pathological aggregation of α-synuclein, called Lewy bodies, resulting in a variety of motor and non-motor symptoms in patients. α-Synuclein is the most studied protein in PD. It does not contain a signal peptide sequence that targets proteins to the secretory pathway, but it can be detected in body fluids such as brain interstitial fluid, CSF and plasma [57, 58].

#### CSF-EVs

Stuendl et al. (2016) unveiled for the first time the presence of α-synuclein in CSF-EVs from patients with PD and dementia with Lewy bodies. They reported a significantly decreased level of α-synuclein in CSF-EVs, isolated through ultracentrifugation, from individuals with PD compared to the control group. Besides, in vitro studies showed the capacity of CSF-EVs from patients to induce oligomerization of soluble α-synuclein in recipient cells [59]. Subsequently, in vivo experiments revealed that the CSF-EVs from α-synuclein-related disorders play a role in the cell-to-cell transmission of α-synuclein to neurons, resulting in the development of α-synuclein inclusion pathology [60]. More recently, CSF-EVs from PD patients have been demonstrated to be capable of inducing α-synuclein aggregation, PD-like symptoms and pathology in healthy mice [61]. Guo and colleagues (2020) demonstrated that the microglial-derived EVs containing α-synuclein play a role in microglial-to-neuron transmission of this protein. Furthermore, CD11b^+^ EVs derived from CSF of PD patients are able to induce α-synuclein aggregation in neurons [62]. Vacchi et al. (2021) showed significantly increased amount of CSF-EVs from PD patients compared to healthy controls [63]. Conversely, Hong et al. (2021) showed no significant differences in the number or size distributions of total EVs in the CSF between PD patients and healthy controls. However, lower proportions of both total α-synuclein and aggregated α-synuclein^+^ EVs were found in PD patients compared to controls [64]. Another significant protein in PD is the leucine-rich repeat kinase 2 (LRRK2). In this regard, LRRK2 has been detected in EVs from biofluids, such as urine and CSF, through EV enrichment by ultracentrifugation and western blot [65]. Then, a method for assessing the overall quantities of LRRK2 was devised, employing targeted liquid chromatography and mass spectrometry, within a clinically feasible 1 ml sample of total CSF [66].

#### Blood-EVs

An approach to identifying PD could be the analysis of proteins, miRNAs, and other factors in blood, which are altered in patients compared to healthy controls. Peripheral CNS-derived EVs in blood have the potential to be biomarkers for PD diagnosis [58] (Table 3).

**Table 3:** CNS-derived EVs in the blood of PD patients

EV-type / Source	PAT / EV-enrichment	EV surface marker	CT	Studied Analytes	Reference
Neuron-derived EVs / Plasma	Plasma / >300 µl 2000× *g* 15 min, 12,000× *g* 30 min + PBS (diluted 1:3)	L1CAM	TEM WB	α-Synuclein	[4]
Neuron-derived EVs / Plasma	Plasma / >300 µl 2000× *g* 15 min, 12,000× *g* 30 min + PBS (diluted 1:3)	L1CAM	-	Tau	[67]
Neuron-derived EVs / Plasma	Serum separator tube, 3000× *g* 15 min 4 °C / 500 µl serum with ExoQuick (System Bioscience) followed by 1500× *g* for 30 min at 4 °C, 1500× *g* for 5 min at 4 °C	L1CAM	TEM WB	α-Synuclein	[68]
Neuron-derived EVs / Plasma	Plasma / >300 µl 2000× *g* 15 min, 12,000× *g* 30 min + PBS (diluted 1:3)	L1CAM	TEM TRPS WB	α-Synuclein	[69]
Neuron-derived EVs / Serum	Serum separator tube / 300× *g* 10 min, 2000× *g* 20 min, 10,000× *g* 30 min	L1CAM	NTA	α-Synuclein, Syntenin-1, Clusterin	[70]
Neuron-derived EVs / Plasma	EDTA Plasma separator tube − 1000x *g* 20 min, 1600x *g* 20 min / >300 µl 2000x *g* 15 min, 12000x *g* 30 min	L1CAM	TEM WB NTA	lncRNAs: Linc-POU3F3, α-Synuclein	[71]
Neuron-derived EVs / Serum	Serum separator tube / 300x *g* 10 min, 2000x *g* 20 min, 10000x *g* 30 min / 100–500 µl + pCBMA beads ON 4 °C	L1CAM	SEM TEM WB	α-Synuclein, Syntenin-1	[72]
Neuron-derived EVs / Plasma	Plasma − 250 µl + 2,5 µl Thrombin 5 min RT, 12,000 rpm 5 min 4 °C / 200 µl + 50 µl ExoQuick 1 h 4 °C, 1000x *g* 30 min	L1CAM	TEM NTA	total IRS-1, p-IRS-1, total P70S6K, p-P70S6K, total tau, total Akt, p-AKT, total Erk, p-Erk	[73]
Neuron-derived EVs / Serum	Serum separator tube − 1500x *g* 10 min / 500 µl serum + 500 µl PBS − 4500x *g* 20 min at 4 °C / ExoQuick (System Bioscience) followed by 1500× *g* for 30 min at 4 °C	L1CAM	NTA TEM Exo-Check Exosome Antibody Array (WB)	α-Synuclein (total and oligomeric), transferrin, SNAP-25, VAMP-2, Syntaxin1A	[74]
Neuron-derived EVs / Serum or Plasma	Silicone-coated serum-collection tubes − 1500x *g* 15 min 4 °C / EDTA Plasma − 1500x *g* 15 min 4 °C / 0.5-1 ml 2000x *g* 10 min 4 °C / ExoQuick (System Bioscience) followed by 1500× *g* for 30 min at 4 °C	L1CAM	ELISA Flow Cytometry MRPS TEM	α-Synuclein	[75]
Oligodendrocyte-derived EVs / Serum or Plasma		MOG			
Neuron-derived EVs / Serum	Serum separator tube − 200x *g* 10 min / 300x *g* 10 min, 2000x *g* 20 min, 10,000x *g* 30 min	L1CAM	-	Syntenin-1, Clusterin, α-synuclein	[76]
Neuron-derived EVs / Plasma	Heparin Plasma − 10 min RT, 1500x *g* 10 min / 500 µl 2000x *g* 20 min 22 ºC, 10,000x *g* 20 min 22 ºC / 500 µl plasma + 500 µl PBS + 150 µl EV precipitation reagent, 10 min RT, 10,000x *g* 5 min 22 ºC	L1CAM	TEM DLS WB	α-Synuclein	[57]
Neuron-derived EVs / Serum	Serum separator tube − 2000x *g* 10 min / 300x *g* 10 min, 2000x *g* 20 min, 10,000x *g* 30 min, 250 µl + pCBMA beads, ON 4 °C	L1CAM	-	α-Synuclein, Syntenin-1	[77]

*PAT* Preanalytical technique, *CT* Characterization technique of EVs, *RT* Room temperature, *ON* Overnight, *pCBMA* Poly carboxybetaine methacrylate

α-Synuclein levels are elevated early in the disease process of rapid eye movement sleep behavior disorder, a high risk of developing PD. With repeated failure of intracellular trafficking from endosomes to lysosomes, the release of α-synuclein into EVs seems to be increased, a process that could occur in PD. Therefore, higher levels of α-synuclein in L1CAM^+^ EVs from blood could be a biomarker at the early onset of the disease and be used to monitor efficacy of therapies that target intraneuronal α-synuclein [4, 58, 71–73, 75, 77]. This is not in concordance with the data on CSF-EVs discussed above, which may be caused by increased rates of α-synuclein transport from the CSF to the blood in PD [59]. Nonetheless, another study reported that the levels of α-synuclein in L1CAM^+^ EVs in serum are decreased in PD patients [68]. The discrepancies may be attributed to the lack of specificity of the L1CAM biomarker, patient selection, heterogeneity of measurement methods, and the enrichment of diverse EV subpopulations from different EV isolation techniques. Besides, misfolded α-synuclein levels in L1CAM^+^ EVs in blood were found to be increased in PD patients compared to controls [57]. On the other hand, Dutta et al. (2021) [75] utilized MOG to isolate putative oligodendrocyte-derived EVs in blood. They observed that these MOG^+^ EVs obtained from patients with multiple system atrophy (MSA), a frequent PD-mimic disease, contained higher levels of α-synuclein compared to both PD patients and control subjects. In addition, the ratio between the concentrations of α-synuclein in MOG^+^ EVs to L1CAM^+^ EVs could potentially serve as a sensitive biomarker for distinguishing between PD and MSA. One interpretation of these results suggests that α-synuclein in MSA tends to accumulate in brain oligodendrocytes. Alternatively, this occurs because MSA is confined to the CNS whereas Lewy body pathology also impacts enteric or other autonomic neurons at an early stage in PD [76].

Another study revealed a significantly higher tau level in L1CAM^+^ exosomes from PD plasma compared to AD, suggesting a more active efflux of tau proteins from the brain to the blood in PD than in AD. This observation suggests that tau levels in L1CAM^+^ EVs could serve as a potential biomarker in combined use with α-synuclein, for distinguishing PD from other mimic diseases and aiding in its diagnosis [67]. Similarly, the levels of phosphorylated insulin receptor substrate 1 (IRS-1) and tau proteins in L1CAM^+^ EVs are higher in PD than in controls [67, 73].

The concentrations of other molecules in L1CAM^+^ EVs, such as the long non-coding RNA Linc-POU3F3, have also been determined. PD patients have increased Linc-POU3F3 concentrations in L1CAM^+^ exosomes and decreased glucocerebrosidase activity in the plasma compared to controls. The Linc-POU3F3 and α-synuclein in L1CAM^+^ exosomes, as well as the glucocerebrosidase activity, are closely associated with the autophagic-lysosomal system implicated in PD pathogenesis. Therefore, they hold promise as valuable indicators to assess the severity of PD [71].

Syntaxin1A and VAMP-2 are part of the SNARE complex, the mediator of neurotransmitter release of synaptic vesicles by neurons. The levels of the two proteins in L1CAM^+^ EVs are decreased in PD patients and inversely correlated with oligomeric α-synuclein levels in L1CAM^+^ EVs. In addition, the oligomeric α-synuclein/syntaxin1A and oligomeric α-synuclein/VAMP-2 ratios can distinguish between PD patients and healthy people. In fact, the oligomeric α-synuclein levels in L1CAM^+^ EVs are correlated with disease duration and clinical severity of PD [74].

### AD

AD is the most common neurodegenerative disorder globally, characterized by the accumulation of abnormal proteins within and on the surface of neurons. These changes are detectable when the disease is advanced and clinically apparent, so there is a need for biomarkers to obtain a sensitive and early diagnosis [78]. The principal contribution was related to the microtubule-associated protein tau [79]. Although remaining controversial, the elevated tau levels in AD could be related to an active secretion rather than a widespread neuronal death [80].

#### CSF-EVs

The first report of the presence of tau and its phosphorylated form (p-tau) in CSF-EVs was by Saman and coworkers (2012) [81]. They demonstrated that a large quantity of CSF-tau in early AD patients is EV-associated. At the same time, the p-tau form displayed apparently low levels of oligomerization that is associated with toxicity. In another study, the levels of CD3, CD4, CD45, CD64, β-APP-cleaving enzyme 1 (BACE1), amyloid-β (Aβ), and APP in CSF-EVs were analyzed by flow cytometry in AD patients compared to controls. The results demonstrated a decrease in tau and APP levels in the CSF-EVs of AD patients, while there was no significant difference in the number of EVs or the levels of the other proteins [82]. The authors associated the reduced tau concentration in EVs with the reported altered distribution of p-tau [83] and the dysregulation of mTOR in AD [84], which potentially disrupts the sorting of tau into EVs. A pilot study of CSF-EVs isolated by an affinity capture method that isolates phosphatidylserine^+^ EVs found enrichment of astrocyte-specific molecules in these EVs from AD compared to mild cognitive impairment (MCI) samples. When compared to the ultracentrifugation and SEC methods, the affinity capture method provides the most enrichment of EV proteins and protein yields compatible to mass spectrometry. However, in this study, the levels of total tau and p-tau did not significantly differ among AD, MCI and healthy controls. This may be attributed to the fact that the phosphatidylserine-negative EVs cannot be isolated by this method [85]. Then, an increase of EV concentration detected by nanoparticle tracking analysis in the CSF from AD patients compared to controls was reported [86]. On the other hand, a study by Joshi P et al. (2014) [87] revealed that microglia possess the ability to transform aggregated Aβ into neurotoxic forms by releasing EVs. This process ultimately leads to compromised neuronal viability and synaptic integrity, highlighting the harmful consequences of EV-mediated microglial processing of Aβ. Furthermore, emerging evidence from in vitro and in vivo studies has indicated the potential involvement of EVs in the prion-like propagation of lesions in AD. This mechanism entails the transmission of misfolded proteins and pathological agents between cells, resulting in the progressive spread of neurodegeneration. The findings underscore the significant role of EVs in facilitating the intercellular transfer of disease-associated factors, contributing to the pathological progression observed in AD [88, 89]. Another study has investigated the role of myeloid-derived EVs in CSF in MCI and AD. These EVs, carrying inflammatory mediators and neurotoxic molecules, could potentially contribute to the myelin damage and neuronal loss observed in these conditions [90]. Utz et al. (2021) [91] analysed CSF-EVs carrying tau, p-tau (Thr181 and Ser202Thr205), synaptophysin, and synaptosomal-associated-protein-25 (SNAP-25). They reported higher percentages of synaptophysin^+^ EVs in the CSF of AD in comparison to other non-inflammatory neurological disease controls.

#### Blood-EVs

As in ALS and PD, L1CAM is the most widely used marker to isolate neuron-derived EVs in AD (Table 4).

**Table 4:** CNS-derived EVs in the blood of Alzheimer’s patients

EV-type / Source	PAT / EV-enrichment	EV surface marker	CT	Studied Analytes	Reference
Neuron-derived EVs / Serum and Plasma	Serum or Plasma separator tube / 500 μl serum or plasma + 500 μl PBS − 1500x *g* 20 min / ExoQuick (System Bioscience) followed by 1500× *g* for 30 min at 4 °C	L1CAM	-	Insulin receptor substrate (IRS-1): total IRS-1, p-IRS-1	[92]
Neuron-derived EVs / Serum and Plasma	Serum or Plasma separator tube / 500 μl serum or plasma + 500 μl PBS − 1500x *g* 20 min / ExoQuick (System Bioscience) followed by 1500× *g* for 30 min at 4 °C	L1CAM	ELISA NTA	Total tau, p-tau, Aβ42	[78]
		NCAM			
Neuron-derived EVs / Plasma	EDTA or heparin plasma separator tube − 2500x *g* 15 min / 500 μl plasma with ExoQuick (System Bioscience) followed by 1500× *g* for 30 min at 4 °C	L1CAM	-	Cathepsin D, LAMP-1, HSP70	[93]
Neuron-derived EVs / Plasma	Plasma sepator tube / 250 μl plasma with ExoQuick (System Bioscience) followed by 1500× *g* for 30 min at 4 °C	L1CAM	TEM NTA	p-tau, Aβ42, NRGN, REST	[94]
Neuron-derived EVs / Plasma	EDTA or Heparin plasma separator tube − 1500x *g* 15 min at 4ºC / 3000x *g* 20 min at 4 °C / ExoQuick (System Bioscience) followed by 1500× *g* for 20 min at 4 °C	L1CAM	NTA ELISA	BACE-1, γ-secretase, sAPPα, sAPPβ, Septin-8, p-tau, Aβ42	[95]
Astrocytes-derived EVs / Plasma		GLAST			
Neuron-derived EVs / Plasma	EDTA or Heparin plasma separator tube − 2500x *g* 15 min / 3000x *g* 20 min at 4 °C / ExoQuick (System Bioscience) followed by 1500× *g* for 20 min at 4 °C	L1CAM	-	Synaptotagmin-2, Synaptopodin, Synaptophysin, Neurogranin, GAP43, Aβ42, p-tau, synapsin 1	[96]
Neuron-derived EVs / Plasma	EDTA plasma separator tube − 2500x *g* 15 min at 4 °C / 3000x *g* 20 min at 4 °C / ExoQuick (System Bioscience) followed by 1500× *g* for 20 min at 4 °C	L1CAM	ELISA	IL-6, TNF-α, IL-1β, C1q, C4b, C3b, C3d, C5b-C9, Factor B, Factor D, Fragment Bb, MBL, CD59, CD46, CR1, DAF, Factor I	[97]
Astrocytes-derived EVs / Plasma		GLAST			
Neural precusor cells-derived Evs / Plasma	EDTA plasma separator tube − 2500x *g* 15 min at 4 °C / 3000x *g* 20 min at 4 °C / ExoQuick (System Bioscience) followed by 1500× *g* for 20 min at 4 °C	CSPG4	ELISA NTA	HGF, FGF-2, FGF-13, IGF-1	[98]
Neuron-derived EVs / Plasma		L1CAM			
Astrocytes-derived EVs / Plasma		GLAST			
Neuron-derived EVs / Plasma	Serum separator tube − 3500 μl 10 min / 10,000x *g* 10 min / ExoQuick (System Bioscience) followed by 1500× *g* for 30 min at 4 °C	L1CAM	WB	SNAP-25	[99]
Neuron-derived EVs / Plasma	EDTA plasma separator tube − 4200x *g* 10 min / 500 μl plasma + 500 μl PBS − 1500x *g* 20 min / ExoQuick (System Bioscience) followed by 1500× *g* for 30 min at 4 °C	NCAM	TEM WB	Aβ42, total tau, p-tau	[100]
Neuron-derived EVs / Plasma	Plasma separator tube / 500 μl plasma + 500 μl PBS − 1500x *g* 20 min / Thrombin 30 min at RT − 6000x *g* 20 min at 4 °C / ExoQuick (System Bioscience) followed by 1500× *g* for 30 min at 4 °C	L1CAM	-	miRNA: miR-212, miR-132-3p	[101]
Neuron-derived EVs / Plasma	Plasma separator tube / 1000–1300× *g* at RT 15–20 min / 500 μl plasma − 3000x *g* 20 min / Thrombin 30 min at RT − 10,000 rpm 5 min at 4 °C / ExoQuick (System Bioscience) followed by 1500× *g* for 30 min at 4 °C	L1CAM	TEM NTA WB	miRNA: miR-23a-3p, miR-223-3p, miR-190a-5p, miR-100-3p	[102]
Neuron-derived EVs / Plasma	EDTA plasma separator tube − 2500x *g* 15 min at 4 °C / 500 μl plasma + 350 µl PBS − 3000x *g* 20 min / ExoQuick (System Bioscience) followed by 1500× *g* for 30 min at 4 °C	L1CAM	TEM NTA WB	Aβ42, p-tau, MMP-9, IL-6	[103]
Brain-derived EVs / Serum	Serum separator tube − 3000x *g* 7 min at 4 °C / 2000x *g* 30 min / 400 μl sample + Total Exosome Isolation reagent (Invitrogen) followed by 10,000× *g* for 10 min at RT	ABCA1	WB ELISA	miR-135a	[104]
Brain-derived EVs / Serum	Serum separator tube − 3000x *g* 7 min at 4 °C / 2000x *g* 30 min / 400 μl sample + Total Exosome Isolation reagent (Invitrogen) followed by 10,000× *g* for 10 min at RT	ABCA1	ELISA	miR-193b	[105]
Neuron-derived EVs / Plasma	EDTA plasma separator tube − 2500x *g* 15 min at 4 °C / 250 μl plasma − 12000x *g* 20 min at 4 °C /+ 175 μl PBS − 1500x *g* 20 min at 4 °C / ExoQuick (System Bioscience) followed by 1500× *g* for 20 min at 4 °C / 3000× *g* for 10 min at 4 °C	L1CAM	TEM NTA WB	Aβ40 and Aβ42	[106]
Neuron-derived EVs / Plasma	Anticoagulant-free tube − 3000 rpm 10 min at 4 °C / 1 ml plasma + Norgen Plasma/Serum Exosome Purification Mini Kit (Norgen Biotek Corp)	L1CAM	WB	miRNA: hsa-let-7e	[107]
Neuron-derived EVs / Plasma	EDTA plasma separator tube − 4200x *g* 10 min / 500 μl plasma + 500 µL PBS − 1500x *g* 20 min / ExoQuick (System Bioscience) followed by 1500× *g* for 30 min at 4 °C	NCAM	TEM WB	Aβ42, Aβ40, total tau, p-tau, miR-384	[108]
		NCAM/ABCA1			
Neuron-derived EVs / Plasma	EDTA plasma separator tube − 4200x *g* 10 min at 4 °C / 500 μl + 500 μl PBS 1500x *g* 20 min at 4 °C / + PBS 1500x *g* 20 min at 4 °C / 200 μl with ExoQuick (System Bioscience) followed by 1500× *g* for 20 min at 4 °C	Amphiphysin 1	TEM NTA WB	Aβ42, Aβ40, total tau, p-tau, miR-29c-3p	[109]
		NCAM/Amphiphysin 1			
Neuron-derived EVs / Plasma	EDTA plasma separator tube / 500 μl NeuroDex total EV isolation reagent (NDX_ESNeuro, Natick, MA) - pellets were resuspended in a binding buffer	GAP43/NLGN3	TEM NTA WB ELISA qPCR	p-tau, Aβ42, proBDNF, syntaxin1, GAP43, GluR2, PSD95, NRGN	[110]

*PAT* Preanalytical technique, *CT* Characterization technique of EVs, *RT* Room temperature

A longitudinal analysis of AD patients showed significant differences in the levels of p-Ser312-IRS-1 and p-pan-Tyr-IRS-1 in L1CAM^+^ EVs as well as the ratio of p-Ser312-IRS-1 to p-pan-tyr-IRS-1 between preclinical and clinical stages up to 10 years before diagnosis, indicating the potential of this blood test to predict the development of AD [92]. Total tau, p-tau and Aβ42 in L1CAM^+^ EVs have been widely used to predict the development of AD up to 10 years before clinical onset. In this sense, it may be possible to identify subjects with a high risk of developing AD in the early stage (diagnosis), and make an accurate prognosis with personalized guidance in their treatment [78]. The upregulation of p-tau and Aβ42 and the downregulation of neurogranin and RE1 silencing transcription factors in L1CAM^+^ EVs could predict the conversion of MCI to AD dementia [94]. Moreover, the levels of cathepsin D, lysosome-associated membrane protein 1, and ubiquitinylated proteins in L1CAM^+^ EVs are significantly higher in AD patients than in healthy controls and patients with frontotemporal dementia, an Alzheimer-mimic disease. In contrast, heat shock protein 70 is significantly lower in AD than in controls. Moreover, these EV proteins are able to classify approximately 96% of patients with AD [93]. Goetzl and coworkers (2016) [96] demonstrated that the levels of synaptophysin, synaptopodin, synaptotagmin-2, and neurogranin in L1CAM^+^ EVs were significantly lower in patients with frontotemporal dementia and AD than in controls. The levels of growth-associated protein 43 and synapsin 1 were decreased only in AD patients. Thus, synaptic proteins may be used as preclinical indicators and progression measurements for senile dementias. Another studied molecule is SNAP-25. AD patients, in contrast to healthy controls, have lower levels of SNAP-25 in L1CAM^+^ EVs [99].

Considering the relation of miRNA deregulation with AD, Cha DJ et al. (2019) [101] demonstrated lower levels of miR-212 in L1CAM^+^ EVs from AD patients than in cognitively intact controls. miR-132-3p in L1CAM^+^ EVs exhibited good sensitivity and specificity for diagnosing AD, but it failed to distinguish AD-MCI from the controls. Significant upregulation of miR-23a-3p, miR-223-3p and miR-190a-5p and significant downregulation of miR-100-3p in L1CAM^+^ EVs of AD patients have been reported [102]. In a larger cohort, the level of let-7e was significantly higher in L1CAM^+^ EVs of AD patients compared to healthy controls [107].

Finally, elevated levels of Aβ42, p-tau, and matrix metallopeptidase 9 (MMP-9) were observed in L1CAM^+^ EVs of AD patients compared to healthy controls, proposing MMP-9 as a promising inflammatory biomarker [103]. Similarly, another study focused on Aβ42 and Aβ40 concluded that Aβ42 levels in L1CAM^+^ EVs reflect amyloid deposition and that the continuous increase of Aβ42 may predict cognitive impairment in patients [106].

On the other hand, other ‘hook’ proteins such as neural cell adhesion molecule (NCAM), GLAST, chondroitin sulfate proteoglycan 4 (CSPG4), and ATP binding cassette subfamily A member 1 (ABCA1) have been explored. Jia et al. (2019) demonstrated in NCAM^+^ EVs that the AD group had significantly higher levels of Aβ42, total tau, and p-tau compared to the amnestic MCI and control groups. They also found a strong correlation of Aβ42, total tau, and p-tau levels in NCAM^+^ EVs between CSF and plasma, showing that plasma samples could have the same diagnostic capacity as their CSF counterparts [100]. Besides, a comparison of L1CAM^+^ and GLAST^+^ EVs showed that the GLAST^+^ EVs present higher levels of BACE1, γ-secretase, soluble Aβ42, soluble APPβ, soluble APPα, glial-derived neurotrophic factor (GDNF), and p-tau compared to L1CAM^+^ EVs in both patients and controls [95]. Furthermore, GLAST^+^ EVs from AD patients show significantly higher levels of BACE1 and soluble APPβ as well as lower levels of GDNF than those from controls. Astrocytes typically play essential roles in supporting the growth and function of neurons, and in pathological conditions such as AD, they can transform into the reactive state that impairs neuronal physiology. In this sense, the same group demonstrated significantly increased levels of complement proteins in GLAST^+^ EVs from AD patients compared to controls, which could potentially damage neurons in the advanced inflammatory stage of AD [97]. Another protein reported by the same group as CNS-derived EV marker is CSPG. They compared a panel of neurotrophic factors in CSPG4^+^ EVs purified from plasma such as hepatocyte growth factor (HGF), fibroblast growth factors (FGF)-2 and − 13, and type 1 insulin-like growth factor (IGF-1). The HGF, IGF-1 and FGF-13, but not FGF-2, show significantly higher levels in the CSPG4^+^ EVs than in the L1CAM^+^ EVs, and all of the four proteins have significantly higher levels than in the GLAST^+^ EVs. Furthermore, the levels of all four proteins in the CSPG4^+^ EVs are significantly lower in patients with mild AD than in cognitively normal control subjects, suggesting that the neurotrophic factors are diminished early in AD [98]. Another protein used to capture and analyse putative CNS-derived EVs is ABCA1. ELISA method is used for ABCA1^+^ EV capture for subsequent miRNA study. miR-135a [104] and miR-193b [105] have been reported to be significantly increased in ABCA1^+^ EVs from the MCI and terminal-stage AD groups in comparison with controls. Then, Li and coworkers (2022) reported, with the same capture method, the possibility of analysing dual-labeled EVs. The levels of Aβ42, Aβ42/40, total tau, p-tau, and miR-384 in NCAM^+^ EVs and NCAM/ABCA1 dual-labeled EVs from individuals with amnestic MCI and AD were significantly elevated compared to those in subjective cognitive decline, vascular dementia, and control groups [108]. Similarly, the levels of Aβ42 and miR-29c-3p in NCAM/amphiphysin 1 dual-labeled EVs from individuals with subjective cognitive decline were compared with those in control and vascular dementia groups [109]. On the other hand, *N*-methyl-*D*-aspartate receptor 2 A (NMDAR2A) is closely related to strengthening of synaptic networks, long-term potentiation, and learning and memory, and can be altered in the presence of cognitive impairment. Tian et al. (2022) [111] reported that the NMDAR2A^+^ EVs could be used as a biomarker to distinguish AD patients from controls, using a flow cytometry-based technology to trace these EVs in the total plasma EVs. Finally, Eitan et al. (2023) [110] developed a novel methodology to immunoisolate putative neuron-derived EVs using a combination of antibodies for growth-associated protein 43 (GAP43) and neuroligin 3 (NLGN3). They showed an enrichment of EV neuronal proteins and highlighted the importance of monitoring preanalytical conditions and incorporating quality control during EV biomarker development for plasma samples from different cohorts. Using three different cohorts, they demonstrated that levels of glutamate receptor 2 (GluR2) and proBDNF, but not mature BDNF, in GAP43^+^/NLGN3^+^ EVs are lower in early AD compared to controls.

### Perspectives and conclusions

**Fig. 2:**
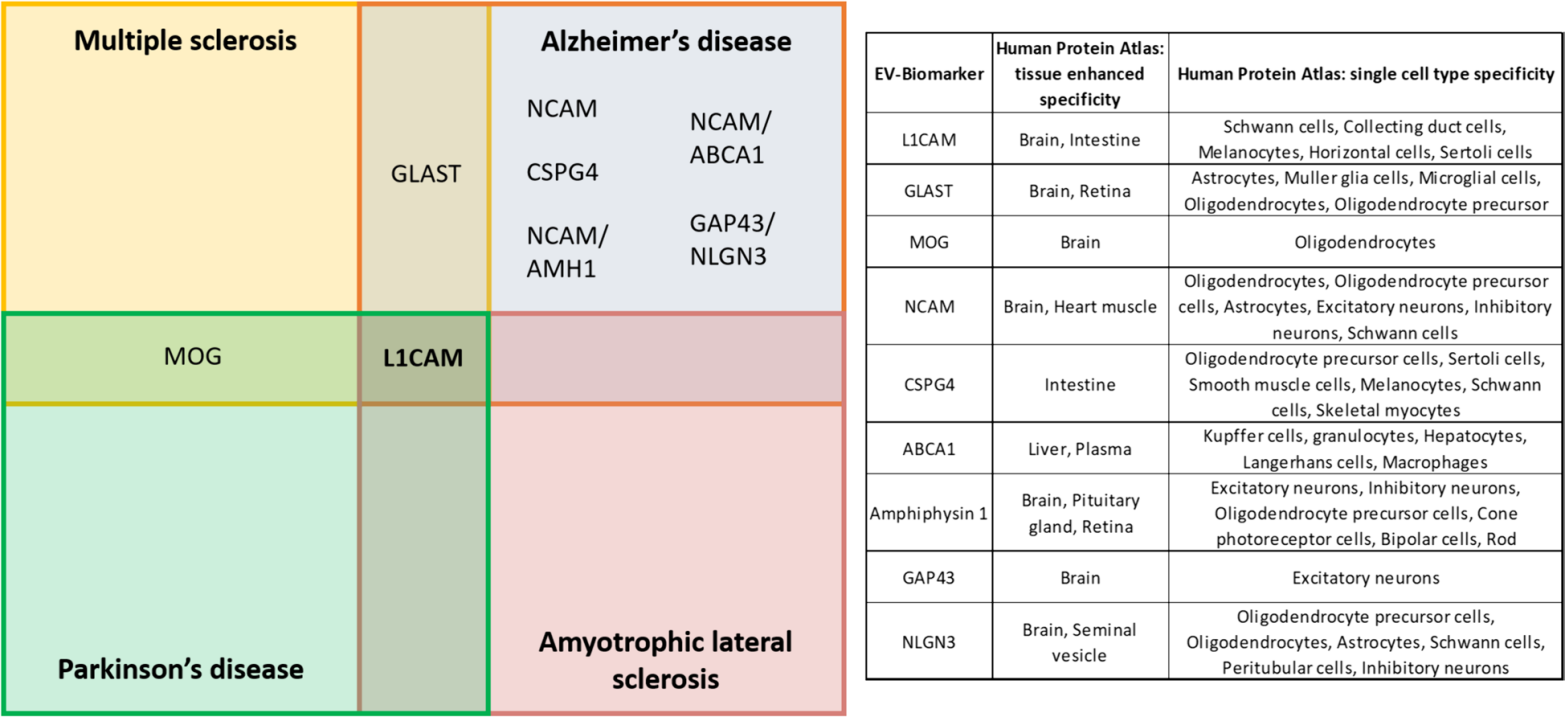
The state-of-art of CNS-derived EVs as circulating biomarkers in blood. Thirty-one out of thirty-nine published papers that reported biomarkers in blood-EVs for MS, ALS, PD, and AD, used L1CAM to isolate putative neuron-derived EVs. Besides L1CAM, others such as GLAST in MS and AD, and MOG in MS and PD, have started to be used. Besides, dual biomarkers such as GAP43/NLGN3 are considered in AD to achieve more specificity. On the other hand, the human protein atlas database can be used to check tissue specificity and cell-type specificity. To note, all the contributions were performed using blood samples from patients with MS, ALS, PD, and AD

The idea of having a window to the processes that are happening in the CNS is attractive and has become an important field of CNS research. Although imaging techniques provide an amazing view and represent powerful tools in the clinic, they do not reach the molecular level. This review covers the main evidence related to EVs in the CSF and blood in order to decipher new CNS-derived biomarkers for MS, ALS, PD, and AD. All reported non-invasive biomarkers to isolate CNS-derived EVs from blood, and an examination of their tissue specificity, are summarized in Fig. 2.

Although blood is the most used and easily obtained biofluid in clinical practice, in recent years, the frontier of the EV isolation has been extended to other non-invasive and easily accessible biofluids such as saliva and tears. Salivary glands, oral mucosal cells and oral flora likely contribute to the composition and origin of saliva-EVs [112]. To mention, levels of oligomeric α-synuclein [113] and its phosphorylated form [114] are significantly higher in saliva-EVs from PD patients than in healthy controls. On the other hand, tears are considered a useful biofluid. Putative neuronal- and microglial-derived EVs were reported, for the first time, in tears from MS patients. In line, CSF- and tear-EVs from MS subjects contain about 70% of the same protein cargo. Besides, both types of EVs present 73.1% of coincidence in significant biological processes related to EV proteins. These authors confirmed an EV-mediated molecular link between CSF and tears, showing the ability of EVs to transmit information from the CNS to a peripheral biofluid [115].

There are two major technical barriers to the research and clinical applications of EVs: (1) simplifying and standardizing the isolation process, increasing the yield or avoiding the need of isolation; and (2) distinguishing among EV subpopulations [116]. While it is impossible to achieve a perfectly pure sample without non-EV particles and other contaminants, it is essential to be able to quantify and exhaustively describe the individual components of an EV preparation. However, traditional methodologies that provide bulk information do not adequately characterize the exact composition of EVs because they inevitably average results from different subpopulations. Thus, more sensitive methods such as single-vesicle analysis could be used to illustrate EV diversity, as recent studies demonstrated [116–118]. ExoView, Single Molecule Array (Simoa), microfluidic chips, and targeted mass spectrometry represent recent breakthroughs in immunoassay technology that are expected to facilitate the application of EV proteins in clinical diagnosis in the near future [85]. In this sense, a crucial task ahead is the exploration and identification of more specific EV biomarkers to analyse CNS-derived EVs in blood. In addition, establishing a panel of EV biomarkers to analyse CNS-derived EVs in blood could increase the specificity and sensitivity necessary for these types of studies. At the same time, and given the heterogeneity of obtained EV samples, an exhaustive and transparent report of the methods using a clear nomenclature, is required to facilitate the interpretation and replication of the experiments [119]. Finally, the presence of inconsistent results highlights the critical need for multicenter studies involving larger cohorts in both discovery and validation groups. Therefore, future research endeavors should prioritize the inclusion of diverse populations that accurately represent Caucasian, African and Asian differences. This approach enhances the robustness and validity of the conclusions.

Summarizing, one approach to advancing into the use of EVs as brain fluid biopsies is to discover new EV surface biomarkers by analysing non-accessible but highly informative samples, such as CSF-EVs, and then translate these findings into a more readily available biofluid with a higher complexity, such as blood, saliva, or tears. Finally, after validation, the EV biomarkers can be directly and specifically traced in the samples, or a targeted EV isolation technique can be used to obtain the putative CNS-derived EVs of interest. In other words, EVs could be one of the most promising candidates to operate as nanosized biological platforms for analysing multidimensional bioactive cargos to mirror brain physiopathology and reflect both localized and systemic changes.

## Data Availability

Not applicable.

## Abbreviations

ABCA1: ATP binding cassette subfamily A member 1
AD: Alzheimer’s disease
ALS: Amyotrophic lateral sclerosis
APP: Amyloid precursor protein
Aβ: Amyloid-β
BACE1: β-APP-cleaving enzyme 1
BCNSB: Blood-CNS barrier
Blood-EVs: Blood-derived EVs
CIS: Clinically isolated syndrome
CNS: Central nervous system
CSF: Cerebrospinal fluid
CSF-EVs: CSF-derived EVs
CSPG4: Chondroitin sulfate proteoglycan 4
EVs: Extracellular vesicles
FGF: Fibroblast growth factor
GDNF: Glial-derived neurotrophic factor
GFAP: Glial fibrillary acidic protein
GLAST: Glutamate aspartate transporter
HGF: Hepatocyte growth factor
IB4: Isolectin B4
IGF-1: Type 1 insulin-like growth factor
IRS-1: Insulin receptor substrate 1
ISEV: International Society of Extracellular Vesicles
L1CAM: L1 cell adhesion molecule
LRRK2: Leucine-rich repeat kinase 2
MBP: Myelin basic protein
MCI: Mild cognitive impairment
MMP-9: Matrix metallopeptidase 9
MOG: Myelin oligodendrocyte glycoprotein
MRI: Magnetic resonance imaging
MS: Multiple sclerosis
MSA: Multiple system atrophy
NCAM: Neural cell adhesion molecule
NMDAR2A: N-methyl-D-aspartate receptor 2 A
PD: Parkinson’s disease
PM: Plasma membrane
PPMS: Primary progressive multiple sclerosis
P-tau: Phosphorylated tau
RRMS: Relapsing-remitting multiple sclerosis
SEC: Size exclusion chromatography
SNAP-25: Synaptosomal associated protein 25
TDP-43: Transactive response DNA binding protein 43
